# Synthesis and biological characterization of silver nanoparticles derived from the cyanobacterium *Oscillatoria limnetica*

**DOI:** 10.1038/s41598-019-49444-y

**Published:** 2019-09-10

**Authors:** Ragaa A. Hamouda, Mervat H. Hussein, Rasha A. Abo-elmagd, Salwa S. Bawazir

**Affiliations:** 1 Department of biology, Faculty of sciences and Arts Khulais, University of Jeddah, Jeddah, Saudi Arabia; 2 Present Address: Department of Microbial Biotechnology, Genetic Engineering & Research Institute, Sadat University, Sadat city, Egypt; 3 Botany department, Faculty of science, Mansoura University, Mansoura, Egypt

**Keywords:** Nanoparticles, Applied microbiology

## Abstract

Using aqueous cyanobacterial extracts in the synthesis of silver nanoparticle is looked as green, ecofriendly, low priced biotechnology that gives advancement over both chemical and physical methods. In the current study, an aqueous extract of *Oscillatoria limnetica* fresh biomass was used for the green synthesis of Ag-NPs, since *O*. *limnetica* extract plays a dual part in both reducing and stabilizing Oscillatoria-silver nanoparticles (O-AgNPs). The UV-Visible absorption spectrum, Fourier transforms infrared (FT-IR), transmission electron microscopy (TEM) and scanning electron microscope (SEM) were achieved for confirming and characterizing the biosynthesized O-AgNPs. TEM images detected the quasi-spherical Ag-NPs shape with diverse size ranged within 3.30–17.97 nm. FT-IR analysis demonstrated the presence of free amino groups in addition to sulfur containing amino acid derivatives acting as stabilizing agents as well as the presence of either sulfur or phosphorus functional groups which possibly attaches silver. In this study, synthesized Ag-NPs exhibited strong antibacterial activity against multidrug-resistant bacteria (*Escherichia coli* and *Bacillus cereus*) as well as cytotoxic effects against both human breast (MCF-7) cell line giving IC50 (6.147 µg/ml) and human colon cancer (HCT-116) cell line giving IC50 (5.369 µg/ml). Hemolytic activity of Ag-NPs was investigated and confirmed as being non- toxic to human RBCs in low concentrations.

## Introduction

Nowadays, nanoscience is a rapidly developing field contributed to produce a wide range of various synthesized metal nanoparticles (MNPs). Owing to the unique physicochemical properties of MNPs and their shapes, a promising scientific area of research appeared for biotechnical applications in biomedicine, environmental bioremediation, optical and electronic fields as well as usage in drug delivery and bioimaging^1,2^. For instance, MNPs possess increased electrical conductivity, roughness and the ability to strength metals and alloys^3^. Silver is the most noble metal in fabrication of nanoparticles due to its wide spectrum of bactericidal and fungicidal activities as well as its ability to coordinate with various ligands and macromolecules in microbial cell. Silver has been widely used in control of microbial proliferation as well as curing wound healing due to its anti-inflammatory effect^4,5^. Usually, coatings containing various silver salts have been applied to inhibit the microbial infections subordinated with medical tools (catheters, wound dressing and orthopedic and cardiovascular implants)^6^. Silver nanoparticles have opened new various disciplines in biomedical protocols, since this marked reactivity of nano-silver was attributed to their larger surface area-to-volume ratios^4^. Production of AgNPs was attained physically and chemically by different approaches where these protocols in spite of producing pure and characterized nanoparticles have some disadvantages as being expensive in addition to their hazard effects on the environment. Generally, biogenic synthesis of silver nanoparticles becomes necessary via green chemistry concepts to produce silver nanoparticles with enhanced stability^7^. Green formation of metal nanoparticles by naturally biodegradable components including polysaccharides, biopolymers, vitamins, plant extracts and microorganisms represent sustainable resources in biosynthesis of metal nanoparticles^8,9^. Subsequently, using microorganisms as bacteria, fungi, microalgae and cyanobacteria in addition to plant extracts and macro algae could induce the required reduction for metal nano synthesis providing an eco-friendly, low priced technology as well as simplicity in scaling up for high production^10^. Plant-based silver nanoparticles synthesis is feasible, ecofriendly as well as possessing catalytic activity for degrading different organic pollutants as azo dyes^11^. Eukaryotic algae as *Chaetocerros calcitrans* and *Chlorella salina* could be used to reduce silver for the formation of nanoparticles^12^. Cyanobacteria have a significant potential among microorganisms for the green synthesis of metal nanoparticles on large scale with various sizes and shapes due to being a sustainable resource for various metabolic products with significant biotechnological applications^13^. Cepoi *et al*.^14^ considered *Spirulina Platensis* and *Nostoc linckia* as nanofactors according to presence of many bioactive substances. Usage of microorganisms enables synthesis of silver nanoparticles through acting as a reducing agent as well as functionalizing nanoparticles surface^15^. Consequently, these multifunctional cyanobacteria significantly decrease reaction stages as well as prevent the need of external stabilizing agents. Shukla *et al*.^9^ used *Oscillatoria willei* NTDM01, the marine cyanobacterium in production of silver nanoparticles where, the reaction mixture of incubating silver nitrate with *Oscillatoria* biomass became yellow within 72 h indicating AgNPs production^8^. Paszkiewicz *et al*.^16,17^ reported that biosynthesis of metallic nanoparticles using marine resources as *Padina pavonia* (brown macro alga) in the bioreduction of silver cations since marine macro algae contain different natural products as alkaloids, lipids and steroids, phenolic compounds and flavonoids, polysaccharides as well as some chemical functional groups (hydroxyl, carboxyl, amino) that act as efficient metal reducing and capping agents in one step^18^.

Currently, bacteria signify a main threat facing medical remediation since the appearance of antibiotic resistant bacterial strains as a consequence of some complicated influences of the evolution and spread of resistance mechanisms^19^. Recently, drug resistance had been emerged as a complicated remediation problem according to the over usage of the antibiotics and drugs in treating infectious diseases in addition to the harmful effects and drawbacks associated with antibiotics as immune-suppression, hypersensitivity and allergic effects. Consequently, developing new antimicrobial drugs for treating microbial pathogens and therapeutic antimicrobial agents of marine plant origin have high remediation effects^20^. The new developing discipline, nanotechnology, stimulated the production of metal NPs especially AgNPs characterized by low toxic influences to human and high bactericidal potential. AgNPs may be used as an alternative to antibiotic drugs exhibiting better effect on multidrug resistant bacteria^21^. The existence of protein caps on nanoparticles support both stabilization and binding to bacterial cell surface leading to increments in binding and absorption of drug on patient cells^22^. The mode of action of AgNPs antibacterial potential is discussed on the basis of disturbing bacterial cell permeability, cellular respiration as well as penetration inside the bacterial cell causing damage via reacting with DNA and protein (phosphate and sulphate containing compounds)^23^.

Nowadays, nontoxicity of the biosynthesized AgNPs when used in low doses become a research interesting issue for medical biotechnology especially in production of non-plant origin polymers, wound recovery and drug delivery^24^. At present the war against cancer are progressively continued including the development of therapies and recovery protocols, where the challenge lies in formulating drugs with high potential anticancer effective compounds with cytotoxic action have been derived from algae^25,26^. Cancer is a complicated very serious genetic disease characterized by uncontrolled and abnormal cells division whereas cancer cells use blood and lymph systems to spread throughout the body^27^. In spite of accepting chemotherapy and radiation therapy as modes of cancer bioremediation, these protocols destroy both normal and cancer cells as well as the usable chemotherapeutic agents resulted in detrimental side effect^28^. In order to overcome these disadvantages a biocompatible, cost-effective protocols, with lowest side effects must be conducted. Nanoparticles as anticancer drug are successfully applied due to their high surface volume ratio and high binding activity which facilitate entrance of cells by diffusion. Consequently it’s commonly to use silver nanoparticles according to their distinctive catalytic, bactericidal, therapeutic activities and stability as well as improvement of nanodevices and therapeutic preparation for diagnoses and treatment of cancer^29^. Nanomedicine is a promising area that could probably induce change affecting cancer treatment protocols, which gives a modern viewpoint for detection, prevention, and bioremediation of tumor^30^. The green synthesized AgNPs are effective anticancer mediators used in evaluation of their cytotoxic effect against different cancer cells *in vitro*^31^. Algal mediated silver nanoparticles synthesis has good potential *in vitro* cytotoxicity investigation in malignant cell culture as human breast (MCF-7) cell line and human colon cancer (HCT-116) cell line^32^. Silver nanoparticles synthesized by macro algae as Turbinaria turbinata and some micro-algae such as *Anabeana oryzae*, *Nostoc muscorum* and *Calothrix marchica* have high cytotoxic effect against Ehrlich ascites carcinoma (EAC) tumor^33,34^. Aqueous extract of the microchlorophyte *Dunaliella salina* can be used for the bioreduction of silver atoms to form AgNPs^35^. Numerous comparison studies were conducted to differentiate between the effects of recognized anticancer drug, cisplatin and algal mediated synthesized AgNPs for treating MCF-7 (cancer) cell lines exhibiting the potency of AgNPs against MCF-7 cancer. Green synthesized AgNPs using Acalypha indica Linn aqueous extract exhibit 40% cell inhibition of human breast cancer cells (MDA-MB-231)^36^. In the same context AgNPs formed by *Taxus baccata* aqueous extract showed effective anticancer potential against MCF-7 cells (IC50 = 0.25 µg/mL) using MTT assay^37^. Antitumor potentiality (cytotoxicity) of both AgNPs and silver cations was expressed via oxidative stress as well as inflammation through production of reactive oxygen species that lead to DNA destruction and mitochondrial membrane potential disorder, releasing cytochrome c and resulting in mitochondrial related apoptosis and necrosis to cell proliferation and carcinogenesis^38^. The antileukemic effect of silver nanoparticles on some leukemic cell lines (K562, MOLT3 and REH) in order to study the biocidal effect on leukemic cell division^39^. Their results demonstrated significant antiproliferative impact of AgNPs synthesized by *Lyngbya majuscula* on the used cell lines in a dose and time independent manner.

This study focused on the green synthesis on AgNPs using *Oscillatoria limnetica* aqueous extract as a reducing and stabilizing agents. The produced AgNPs would be characterized using UV-Visible spectroscopy, FT-IR, SEM and TEM techniques. Furthermore, the antimicrobial activities of silver nanoparticles synthesized would be evaluated against different human pathogens as well as hemolytic activity and cytotoxic effects against both human breast (MCF-7) cell line and human colon cancer (HCT-116) were investigated.

## Results and Discussion

### Biosynthesis and UV-visible spectroscopic profile of synthesized O-AgNPs

**Figure 1:**
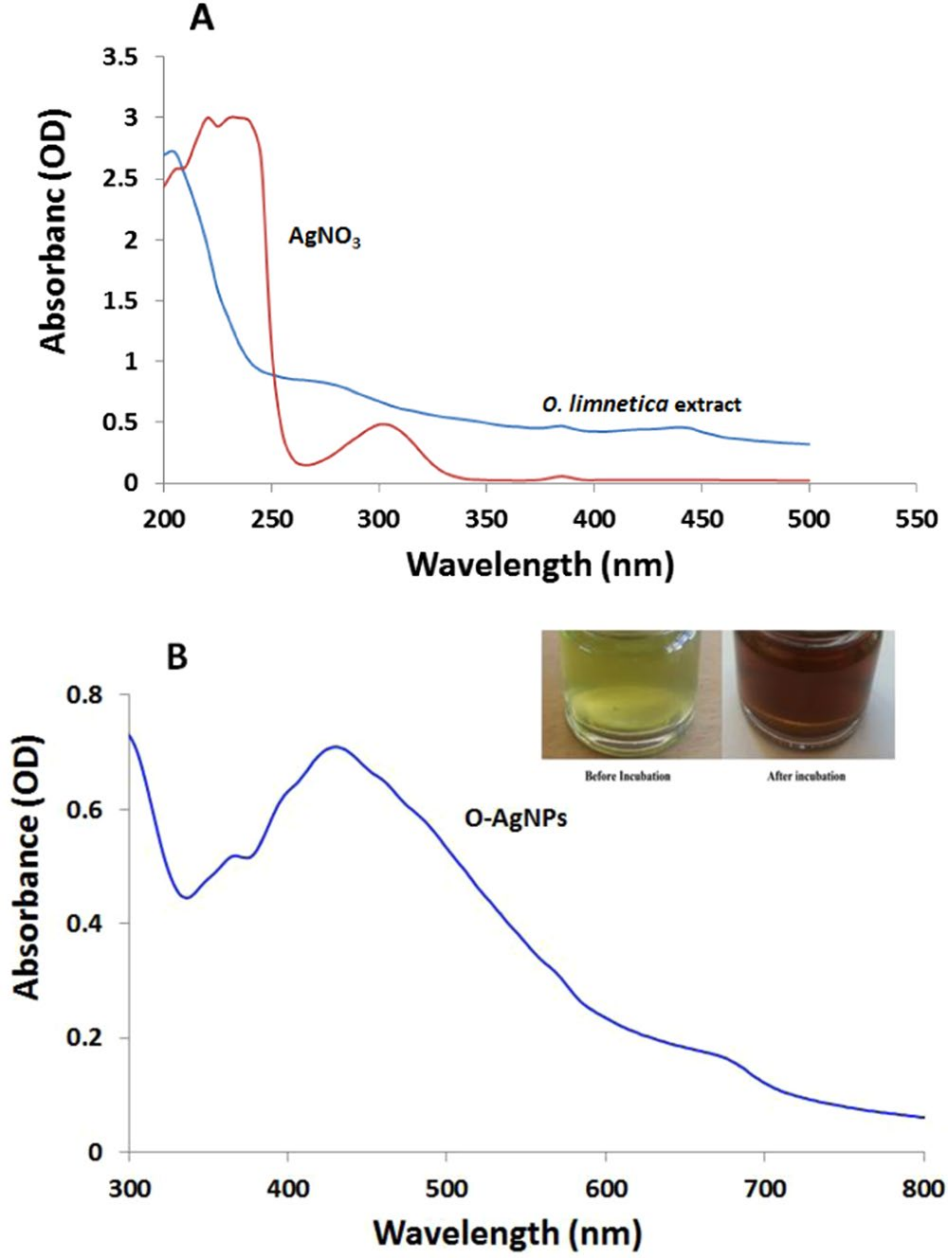
UV-visible absorption spectrum of *O. limnetica* extract & AgNO_3_ (**A**) and silver nanoparticles (O-AgNPs) biofabricated after 48 h of reaction (**B**).

The biofabriction of AgNPs could be ascertained using a visual marker represented by chromatic change of the reaction substrate, since the color transition from green to brown imply the biotransformation of Ag^+^ ion to Ag^0^ indicating synthesis of silver nanoparticles^40,41^. Figure (1) illustrated the formation of O-AgNPs surface plasmon peak at 426 nm with intensity of 0.707 nm at the addition of 0.5 mM AgNO_3_ to *O*. *limnetica* aqueous extract^42–44^ where there is no absorption band was observed neither with algae extract nor with AgNO_3_ solution. Nanometals showed conspicuous spectral characteristics according to the surface plasmon resonance (SPR) due to mutual vibrations of the free electrons resonance with light wave which influenced by each of size and shape of the synthesized NPs^45,46^. Consequently, broadening of the SPR peak width is considered an agreeable detector of the nanometal size and its Polydispersity, where the range of 320–580 nm is characteristic λmax for AgNPs biofabriction as mentioned by Govindaraju *et al*. and Jena *et al*.^12,41^, where frequency and band width of SPR is not only depending on both size and shape of the metal nanoparticles but also on the dielectric constant characterized the metal itself as well as adjacent medium^47^. Many studies have been reported the potentiality of various cyanobacteria crude extracts for synthesizing silver nanoparticles^15,39,48^. Mahdieh *et al*.^49^ suggested the involvement of a two- step mechanism in which firstly there was adherence of the aqueous metal ion to the surface of algal cells as a result of the electrostatic attraction between the positively charged metal ions and negatively charged carboxylate ions present on the surface of cells. This is followed by reduction of ions to metal nanoparticles due to the secretion of cellular reductases by algal cells. Light-mediated protocols for NPs synthesis are dependent on reduction of metal cation Mn^+^ to Mn^0^ either by direct or indirect via photosystem II^50^. In addition Sathishkumar *et al*.^48^ suggested the involvement of metabolites as saponins, quinines, flavonoids and terpenoids in the bioreduction of silver nanoparticles. The current result (Fig. 1) may be interpreted on the basis of reducing effect of proteins, enzymes, metabolites like flavonoids which quantified to 89 ± 11 µg/ml in the *O*. *limnetica* crude extract and /or phycobilisomes with its content of phycobiliproteins and chlorophyll a rich thylakoids that mediated the conformational changes to absorb light energy for initiating photosynthesis. After absorbing light, chromospheres excited molecules from the ground state to an electronic excited one^51^. Consequently under illumination, AgNO_3_ may be reduced in the reaction mixture as a result of electrons that jump between energy levels forming silver nanoparticles. Ali *et al*.^52^ used *Oscillatoria Willei* NTDM01 extract in reduction of silver ions forming spherical AgNPs ranging from 100–200 nm in size, indicating the role of protein molecules as a capping agent in the synthesis process. Moreover, Shankar *et al*.^53^ suggested that proteins combined to the nanoparticles either through free amine groups or cystein residues, indicating the acquirement of microalgae in the fabrication of silver nanoparticles^41^. Whereas, the size, number and shape of the produced NPs are reliant on the concentrations and the exposure time to silver ions^54^ using Spirulina platensis. For optimizing the O-AgNPs synthesis the following effective factors must be investigated.

### pH of the synthesis medium

**Figure 2:**
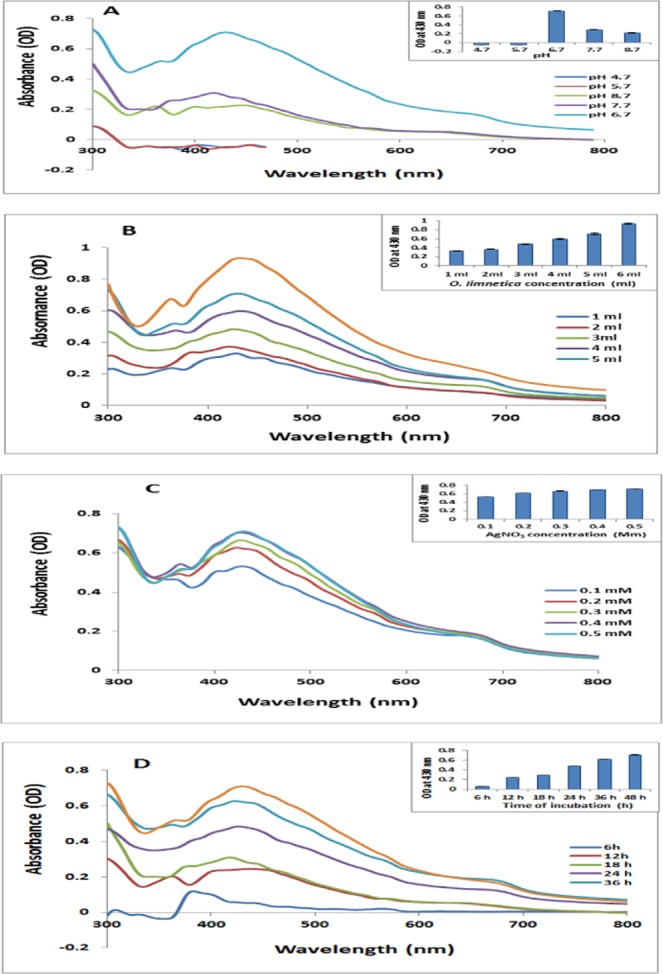
(**A**) UV-visible absorption spectra of O-AgNPs biosynthesized at various pH values [4.7, 5.7, 6.7, 7.7 and 8.7]. (**B**) O-AgNPs at various *O. limnetica* extract content (ml) [1, 2, 3, 4, 5 and 6]. (**C**) O-AgNPs at various AgNO_3_ concentrations (mM) [0.1, 0.2, 0.3, 0.4 and 0.5]. (**D**) UV-Visible spectra of O-AgNPs at different time intervals (h) [6, 12, 18, 24, 36 and 48].

pH value greatly influence the size and morphology of the biologically synthesized nanoparticles, where pH strongly altered the electrical charges of biomolecules and capping agents resulting in changing their ability to bind and reduce metal ions^55,56^. In this study, the effect of pH on the formation of AgNPs was investigated over these values 4.7, 5.7, 6.7, 7.7, and 8.7. Coloration of the reaction mixture as well as peak intensity were pH dependent as appeared in case of pH 4.7 and 5.7 since a colorless solution without any characteristic peak was observed (Fig. 2A). At pH 6.7, 7.7 and 8.7 various brown coloration were demonstrated and the synthesized O-AgNPs showed peaks intensities 0.710, 0.309 and 0.227 nm at wavelengths 430, 418 and 444 nm respectively, indicating the optimum pH at 6.7. Rajesh *et al*.^57^ interpreted the change in peak intensity according to the presence of large particles that in need of less excitation energy. In support of the present results Gan *et al*.^58^ who reported that at slightly acidic pH a large number of functional groups being available for silver binding facilitated a higher number of Ag ions to bind and subsequently form a large number of nanoparticles with smaller diameters.

### Effect of *O*. *limnetica* extract

Concentration of the bio-reducing agents is one of the significant parameters that affect characteristics of NPs through controlling its size and shape. Figure (2B) showed that increasing concentration of *O*. *limnetica* (1, 2, 3, 4, 5 & 6 ml) induced apparent increasing in the absorbance of the resulted peaks (0.330, 0.373, 0.484, 0.599, 0.710 & 0.934) respectively indicating high production of O-AgNPs^8^. In addition to shifting in the position of spectral peaks from 420 to 430 nm resulted in increasing broadening of peaks owing to increase in particles size. These observations may be attributed to the high reducing capacity of *O*. *limnetica* extract that encourages the secondary reduction of Ag ions^59^ and/or to the excitation of surface plasmon vibrations in the silver nanoparticles^60^.

### Effect of silver nitrate concentration

Figure (2C)^42,61^. The maximum absorbance (0.710 nm) was recorded illustrated surface plasmon resonance (SPR) with absorption bands ranged from 422 nm to 430 nm with gradual increasing in intensity ranged from 0.531 nm to 0.710 nm in silver nitrate dose responding manner which confirmed the formation of O-AgNPs with 0.5 mM AgNO_3_.

### Effect of reaction time

**Figure 3:**
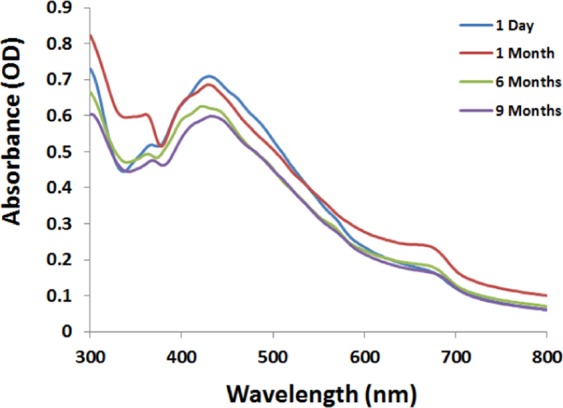
Stability of O-AgNPs.

Biosynthesis of AgNPs has been proved to be a time dependent process^56^. Figure (2D) illustrated the UV-vis spectra of the reaction mixture as a function of time (48 hours) of the process. It was obvious that adequate formation of O-AgNPs started after 18 hours of reaction giving silver surface plasmon resonance band in the range of 418–430 nm with progressive increasing in intensity reaching maximum after 48 hours. The dispersion stability of the O-AgNPs with increasing the storage time demonstrated no significant change in color. UV-visible spectroscopy illustrated shift in the peak position of the surface plasmon resonance from 430 to 432 nm with decreasing in optical intensity from 0.710 to 0.599 nm (Fig. 3) after 9 months.

### Characterization of the synthesized O-AgNPs by electron microscopy

**Figure 4:**
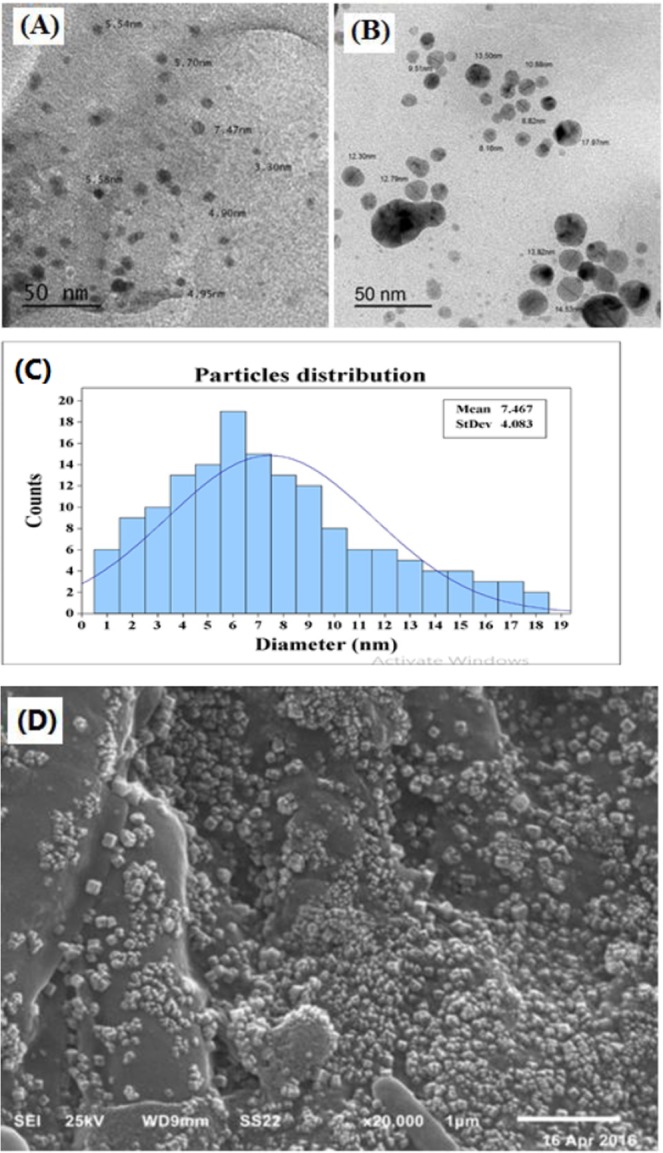
TEM image of O-AgNPs (**A**,**B**), particles size distribution (**C**) and SEM image of biosynthesized O-AgNPs (**D**).

The TEM micrographs (Fig. 4) illustrated the morphology and size of the biofabricated O-AgNPs which are quasi-spherical in shape as well as being anisotropic in nature with diversed size ranging from 3.30–17.97 nm. Moreover, the biosynthesized nanoparticles were well dispersed without significant agglomeration or morphological variations. The present data are in agreement with Singh *et al*.^35^ who developed photo-induced, ecofriendly, low cost method for biofabriction of stable silver nanoparticles from aqueous extract of the micro alga *Dunaliella salina* which performing both reducing and stabilizing effect. In the same context, Abdel-Raouf *et al*.^17^ used *Padina pavonia* aqueous extract in production of AgNPs that characterized by small size and various shapes. The present results were in accordance with that of plaza *et al*.^62^ who initiated their discussion on the chemical composition of the brown algae which had many natural functional components (terpenes, alkaloids as well as amino and fatty acids) functioning as stabilizing agents to prevent aggregation of nanoparticles. SEM images (Fig. 4D) illustrated roughness contour of the synthesized O-AgNPs and good distribution of the precipitated silver nanoparticles^11^.

### FT-IR analysis of the synthesized O-AgNPs

**Figure 5:**
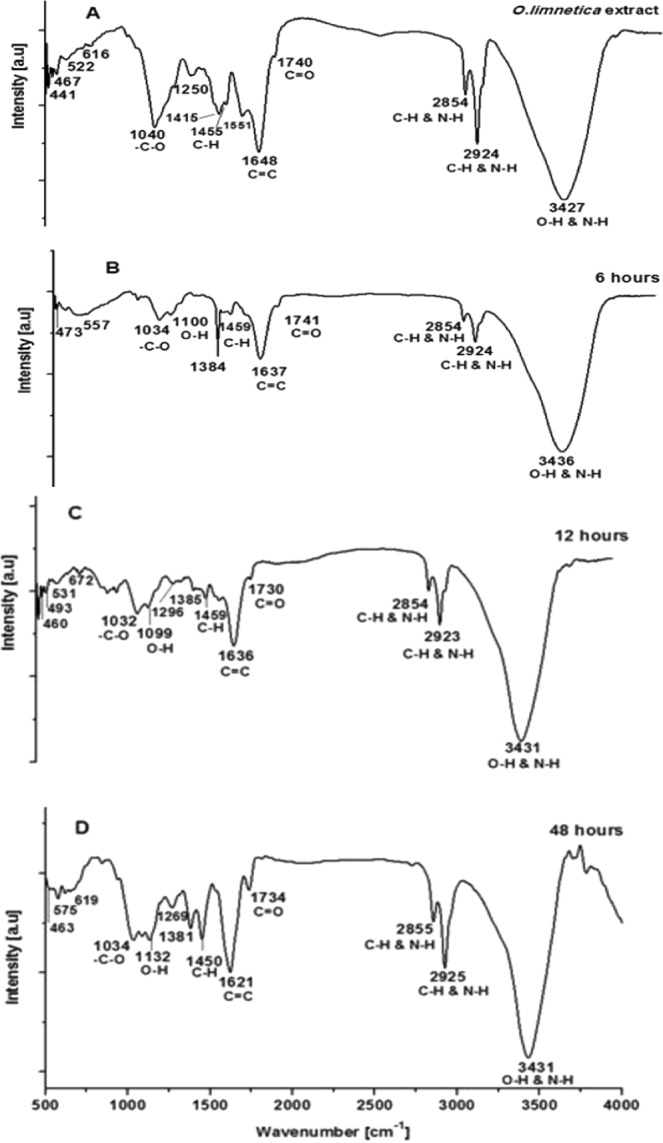
FT-IR spectrum of *O. limnetica* extract and O-AgNPs at different reaction time (h) [6, 12, and 48].

FT-IR measurements were conducted to reveal the possible potential biomolecules that participated in the bioreduction of silver and stabilization of AgNPs^63^. FT-IR profile (Fig. 5A) illustrated 14 peaks positions at 3427, 2924, 2854, 1740, 1648, 1551, 1455, 1415, 1250, 1040, 616, 522, 467 & 441 cm^−1^ for *O*. *limnetica* extract whereas the AgNPs reaction mixture at the end of 6 hours showed 11 spectral bands (Fig. 5B) at the following wave numbers 3436, 2924, 2854, 1741, 1637, 1459, 1384, 1100, 1034, 557 & 473 cm^−1^. Concerning FT-IR analysis of AgNPs reaction mixture after 12 hours (Fig. 5C) elucidated 14 absorbance peaks sites at 3431, 2923, 2854, 1740, 1636, 1459, 1385, 1296, 1099, 1032, 672, 531, 493 & 460 cm^−1^ while the AgNPs preparation (48 h incubation) exhibit 14 spectral bands having the following wave numbers 3431, 2925, 2855, 1734, 1621, 1450, 1381, 1269, 1132, 1034, 619, 575 & 463 cm^−1^ (Fig. 5D). The absorbance bands located between 3000–3600 cm^−1^ assigned to the stretching vibrations of hydroxyl groups and amine groups where N-H is characterized by lower wave numbers than that of O-H^64^. Figure (5) illustrated distinctive broad spectral bands at around 3427–3436 cm^−1^ are characteristic to the O-H stretching vibration type of hydroxyl functional group in polyphenols and N-H stretching vibrations in primary and secondary amines of amino acids, peptides and proteins^65,66^. The peak shifting in AgNPs spectral profile could be attributed to the interactions between those chemical functional groups and AgNPs^67^. It was documented that protein molecules cooperate with AgNPs via free amide groups^68,69^. FT-IR data demonstrated that the amide linkage of the protein possessed the higher potential to join silver and consequently forming protein covering around AgNPs to prevent agglomeration and thereby stabilize the medium^70^. In this study the involvement of hydroxyl groups in the bioreduction process could be confirmed through the shift of the deformation vibration of O-H positioned at about wave number 1100 cm^−1^ in the three AgNPs preparations as well as the observed shift from 1100 to 1132 cm^−1^ which did not appeared in *O*. *limnetica* crude extract^71^.

Presence of spectral peaks at 2924, 2854 and 1455 cm^−1^ may be attributed to aliphatic C-H stretching vibration of hydrocarbon chains and N-H bending vibration^65^. The spectral vibration of aldehydic group (C=O) was illustrated by wave length 1740 cm^−1^ ^72^ while the peak at the range 1648–1621 cm^−1^ could be attributed to amides (N-H) stretching in addition to peptide bond and C=C stretching which may involve in stabilizing nanoparticles by proteins as explained by Castro *et al*.^73^, who investigated the biosynthesis of AgNPs using aqueous extract of either the red macro alga Chondrus crispus or the green alga *Spyrogira insignis*. Spectral band at 1384 cm^−1^ which appeared only in the three AgNPs preparations could be designated to the residual amount of AgNO_3_^74^. Data revealed the presence of absorption bands at 1034 cm^−1^ which may have been attributed to vibration of the -C-O group^75^. Meanwhile the presence of peak at 1450 cm^−1^ is attributed to the vibration of proteins as being stabilizing agent via free amine groups or cystein groups^54^. The peaks positioned at wave numbers 1551 and 1415 cm^−1^ in the IR profile of *O*. *limnetica* extract and disappeared in the three AgNPs preparations. Spectral peaks at 616–672, 522–575 & 441–493 cm^−1^ indicated the bending region of the aliphatic chain. Moreover, bands assigned at 1381, 1269, 1132 & 1034 cm^−1^ can be attributed to either sulfur or phosphorus function groups, which possibly attach silver and perform both capping and stabilizing process of nanoparticles^73^.

### Antibacterial potentiality of O-AgNPs

**Figure 6:**
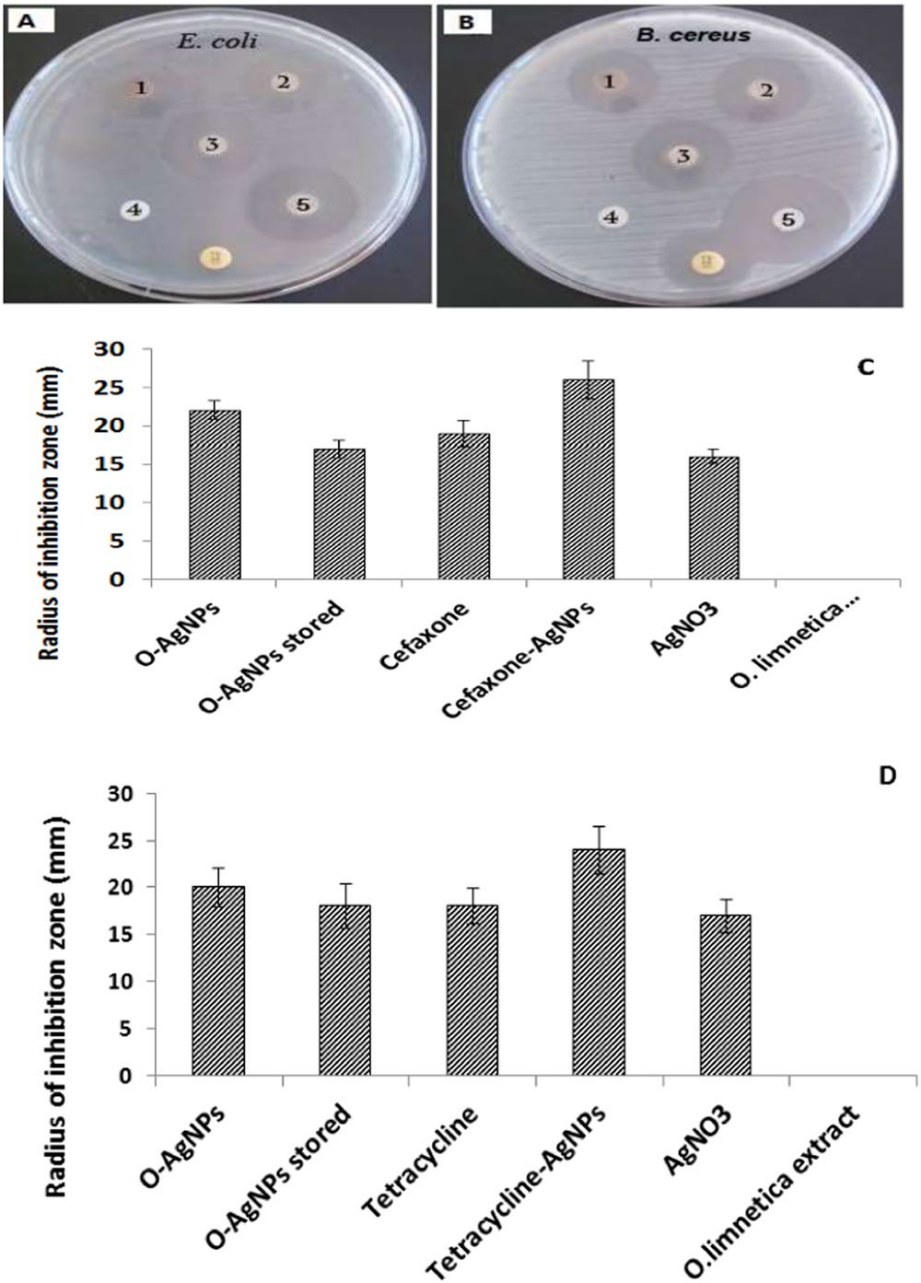
Antibacterial activity (zone of inhibition) of O-AgNPs against two human pathogenic bacteria *E*. *coli* (**A**,**C**) and *B*. *cereus* (**B**,**D**) where (1) is AgNO_3_, (2) O-AgNPs stored, (3) O-AgNPs, (4) is *O*. *limnetica* extract, (5) **A** is Cefaxone-AgNPs & (5) **B** is tetracycline-AgNPs and (6) **A** is Cefaxone & (6) **B** is tetracycline.

Owing to the observed resistance of many pathogenic bacteria towards the already used antibiotics, exploiting AgNPs may be another urgent choice for controlling proliferation of bacterial human pathogens^76^. For evaluating the antibacterial activity of O-AgNPs against *E*. *coli* as well as *B*. *cereus* disc-diffusion method was used, in which zone of inhibition around the holes denoted as a function in bacterial growth inhibition (Fig. 6). It is found that the concerned bactericidal effect may be attributed to Ag^+^ ^77,78^. Jun *et al*.^79^ who referred this antibacterial action to the binding potentiality of Ag^+^ ions with various bacterial cell compartments as DNA molecules and cytoplasm which outflow from the injured cell wall. In the present study the appearance of clear inhibitory zones confirmed the complete growth inhibition of either *E*. *coli* or *B*. *cereus*. The inhibition potential of the freshly prepared O-AgNPs (22 mm against *E*. *coli* and 20 mm against *B. cereus*) was more relatively pronounced to that induced by the tested antibiotics (19 mm for Cefaxone and 18 mm for tetracycline), whereas *O*. *limnetica* extract had no inhibitory effect on the two used bacterial pathogens (Fig. 6). Concerning *E*. *coli*, the maximum antibacterial potential was recorded to the synergistic effect of Cefaxone-conjugated O-AgNPs resulted in 26 mm inhibition zone diameter, which was comparable to the effect of Tetracycline-conjugated O-AgNPs (24 mm) on *B*. *cereus*. Biosynthesized silver nanoparticles exhibit pronounced antibacterial action towards E. coli (Gram negative) more than *B. cereus* (Gram positive). The current results are in accordance with the findings of Sondi *et al*.^80^ about the broad spectrum response of the biosynthesized silver nanoparticles as well as the results of Shanmuganathan *et al*.^70^ who reported that Cefaxone-conjugated AgNPs exhibited significant bactericidal action (23 mm inhibition zone) than the free AgNPs (18 mm inhibition zone). They recommended that these results might be effective in antibiotic-resistant human pathogens.

**Figure 7:**
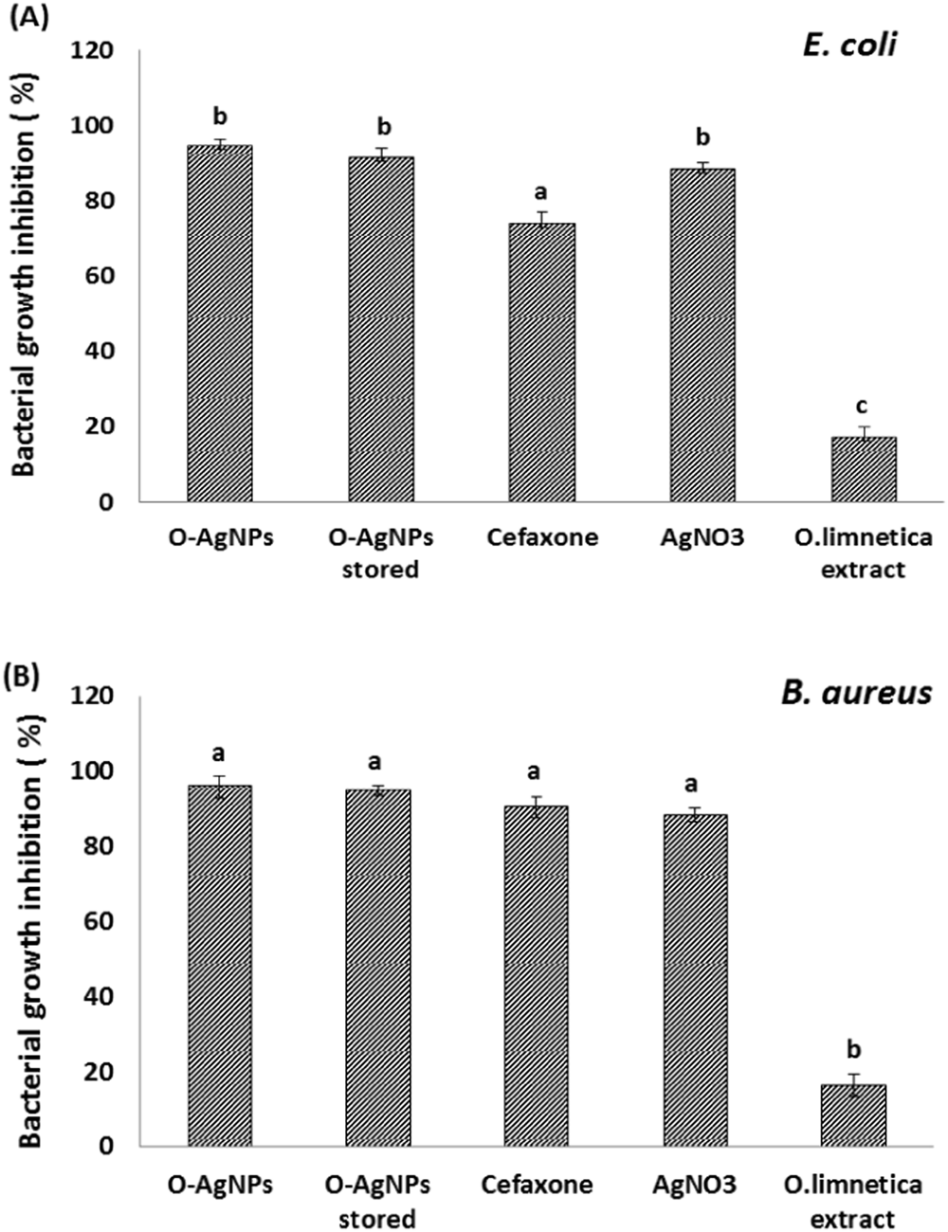
Bacterial growth inhibition (culture optical density) of O-AgNPs against *E*. *coli* (**A**) and *B*. *cereus* (***B***).

**Figure 8:**
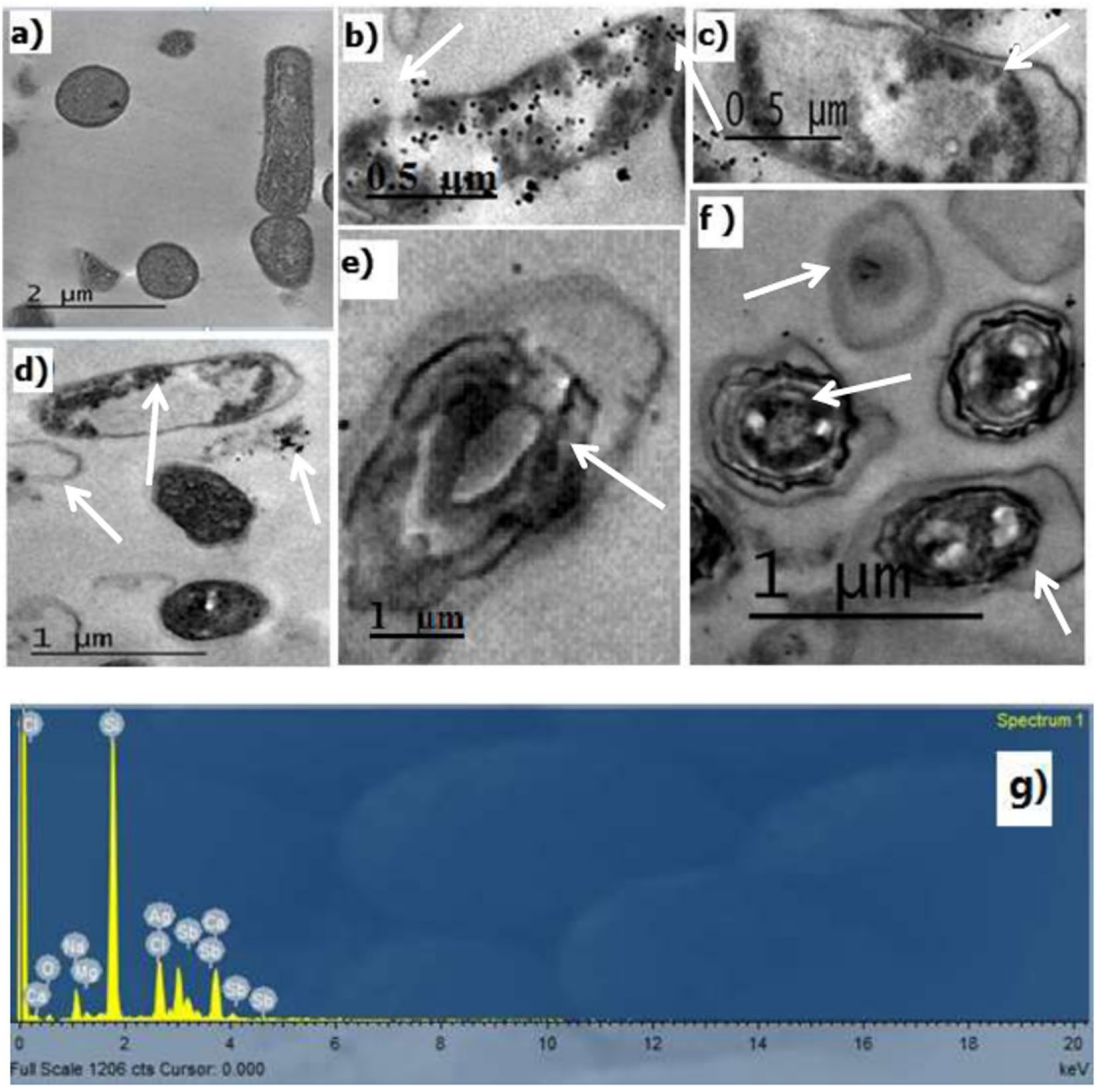
TEM morphological changes of *B. cereus* as cell structure with or without O-AgNPs. (**a**) Structure of intact cell (control), (**b**) damaged cell membrane and releasing of cytoplasm, (**c**,**d**) dark mineral particles and electron dense particles within cytoplasm, (**d**,**f**) cellular disintegration and (**e**,**f**) shrinking of protoplasm and detachment of cellular membrane (cellular deformation). (**g**) EDX spectrum of *B. cereus* cells with O-AgNPs.

On the other hand, *O*. *limnetica* aqueous extract induced inhibitory effect on growth of the two tested bacterial pathogens *E*. *coli* and *B*. *cereus* in LB broth with magnitude of response 16.1% and 17.2% growth inhibition, respectively. In general, the tested silver nanoparticles induced a significant inhibitory response on both tested bacteria at comparison with cefaxone represented by 94.6% and 95.9% growth inhibition for *E*. *coli* and *B*. *cereus*, respectively (Fig. 7). In addition, to compare the effect of AgNPs with ionic Ag^+^, a control test was applied using AgNO_3_ solution which exhibited lower growth inhibition than AgNPs. The magnitude of response recorded as 88.49% & 88.38% bacterial growth inhibition for E. coli and *B*. *cereus*, respectively (Fig. 7). Using disc diffusion method, the inhibition zone around disc was directly proportional with AgNPs concentration which considered a function in toxicity^48^. As a result, their bactericidal effect was attributed to the releasing of Ag^+^ ions from the surface of O-AgNPs. Therefore, AgNPs distribution through solid medium could somewhat reduce its virulence and toxicity as well. In case of liquid medium, Prabhu *et al*.^81^ explained the high antibacterial potentiality of AgNPs after the fact that increasing surface area lead to increase in AgNPs toxicity, where this potent effect may be a result of good dispersion and contact of the elevated silver level with bacterial cells more than in solid medium. In this study, Fig. (8) illustrated the TEM images and elemental analysis of *B*. *cereus* treated with O-AgNPs. Figure (8a) demonstrated the structure of intact cell without O-AgNPs treatment (control), while Fig. (8b) showed damaged cell membrane and leakage of cytoplasm exterior to cell and internal defusing of O-AgNPs to cell. Dark mineral particles and electron dense spots were observed within cytoplasm (Fig. 8c,d). Cell disruption and disintegration (Fig. 8d,f) as well as shrinking of protoplasm and detachment of cellular membrane (Fig. 8e,f) were also observed. Figure (8g) illustrated EDX profile of *B*. *cereus* treated with O-AgNPs which verifying presence of Ag through the optical peak observed approximately at 3 keV showing weight percentage 18.57 and atomic percentage 5.70. According to results of the morphological and elemental analysis (Fig. 8) the mechanism of O-AgNPs in bacterial inactivation may be attributed to damage of cell wall and plasma membrane as a result of protein inactivation and peroxidation of membrane lipids which disrupted the membrane structural integrity resulted in transport disorders and potassium leakage^82^. TEM images demonstrate that O-AgNPs are located at both the surface of the plasma membrane and distributed through the bacterial cells as well. Gram-positive bacteria is characterized by hard cell wall structure formed of a thick peptidoglycan layer that constructed from polysaccharide linear chains having short peptides as cross linkages giving more rigidity structure resulted in difficulties with AgNPs penetration^83,84^. Silver nanoparticles inside the cell may interact with the signaling pathway of bacterial growth using the modulation of tyrosine phosphorylation of recognized peptide substrates, essential for cell proliferation which may aggregates without any contact between the AgNPs, demonstrating its stabilization by a capping agent^83,84^. The surface plasmon resonance has a main part in estimation of absorption spectra of metal nanoparticles, that expands toward longer wavelength with increase in particle size which suggests that AgNPs possessing a large surface area to come in contact with the bacterial cells and hence, it will have a higher percentage of interaction than bigger particles. Moreover, it was noticed that generally there was no differences in the size of O-AgNPs interacting the membrane and inside the cell which showed that O-AgNPs could be transported across the membrane getting inside the bacterial cell, where the size of AgNPs greatly influence its antibacterial potential^85^. The electronic effects can be generated according to the presence of small sized metal particles about 5–20 nm which are exhibited as variations in the electronic structure of the surface, therefore the concerned influence could promote the nanoparticles surface reactivity i. e., the bactericidal effect of silver nanoparticles is size dependent^86^. In the same context, the binding force between the metal particles and the bacteria depends on the surface area of interaction. Sondi *et al*.^80^ studied the penetrating mechanism of nanoparticles into *E*. *coli* subjected to AgNPs and reported that the effects of AgNPs on plasma membrane structure resulting in the development of pits which may be responsible for considerable increments in the membrane permeability influencing transportation across cell membrane. This may be an acceptable interpretation for the entering of AgNPs inside bacterial cell. This explanation may elucidate the biocidal mechanism of AgNPs. Taking the theory of hard and soft acids and bases, it is found that there is a high affinity for silver to combine with either phosphorous or sulfur components of the bacterial cell as previously^87^. Bacterial plasma membrane comprises a lot of sulfur containing proteins which may represent suitable sites for AgNPs attraction where, inside the bacterial cell AgNPs will prefer to combine with proteins containing sulfur and other cell components containing phosphorous as DNA^79^. The alteration of the bacterial membrane and the damages in the DNA which may be produced by AgNPs will influence bacterial metabolic processes as cell respiration which may be blocked in combination with oxygen and sulfhydryl (S H) groups on the cell wall, resulting in consumption of ATP and consequence cell death. For more explanation, silver nanoparticles may discharge Ag^+^ that increased its biocidal activity^79^. These silver ions form a low molecular weight region central to the bacterial cell forming a defense mechanism enabling bacteria to conglomerate DNA for protection against toxins^85^. The surface of bacteria is negatively charged according to the dissociation of excessive carboxylic groups in protein part of the membrane which interact with Ag^+^ leading to enhance the bactericidal effects of AgNPs^50^.

### Hemolytic assay of oscillatoria limnetica- mediated AgNPs

**Figure 9:**
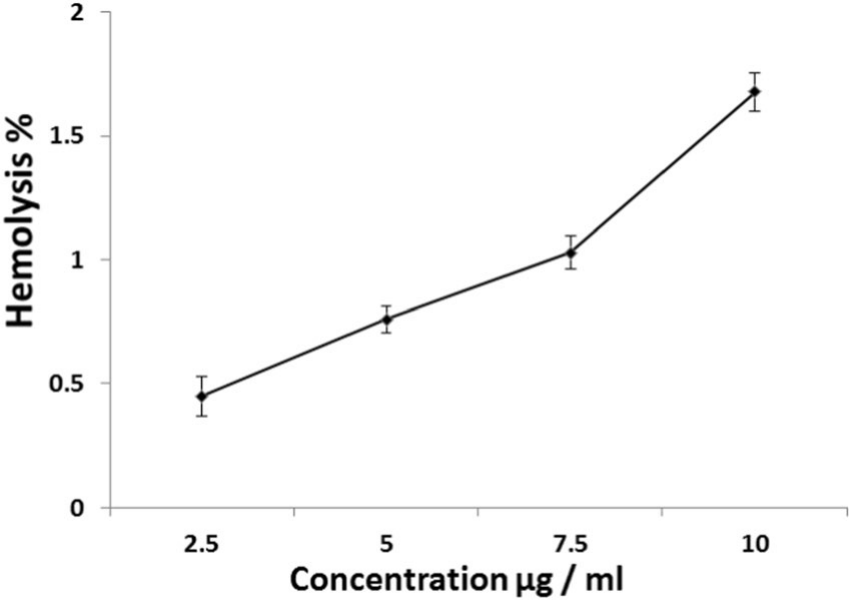
Percentage of hemolysis caused by O-AgNPs.

With increasing demand on clinical employments of silver nanoparticles, where it is vital to ensure their bio safety. Hence, the assessment of hemolytic potential of the used AgNPs is necessary in case of blood-contacting medical devices. Physicochemical characterization of AgNPs and their biocompatibility estimation have to be in consideration for materials used in these devices. The characteristic features of AgNPs, including size and surface area, interfere with its hemolytic activity. Hemolysis occurred when the membrane of the erythrocytes turned into compromised one resulting in haemoglobin leakage which linked with harmful health conditions^88^. The biogenic O-AgNPs were used to investigate its Cytotoxicity on human erythrocytes integrity which was determined by assessment of haemoglobin release spectrophotometry (Fig. 9). The observed results demonstrated an induced hemolytic action on the erythrocytes as a function of increasing O-AgNPs concentration. Data showed cell lysis as 0.45, 0.76, 1.03, 1.68% corresponding to 2.5, 5.0, 7.5, 10 µg/ml O-AgNPs concentrations, respectively. Triton X-100 (+ve control) and PBS buffer (^−^ve control) exhibited 98.64% and 0.011% hemolysis, respectively. For explaining the mode of action of TTX-100 hemolytic potentially, firstly the inclusion of TTX-100 molecules into RBCs membrane resulted in reducing the molecular packing which leads to an increase in permeability causing haemoglobin leakage. Afterward, the membrane was fully solubilized^89^. Mode of action of Ag-NPs for inducing hemolysis still not clearly revealed. The metallic silver undergo ionization when came in contact with the body fluids, releasing Ag^+^ according to particle surface area response^5^. RBC hemolysis *in vitro* could be induced by low concentrations of Ag^+^ ^90^. In biological systems, the quick binding of Ag^+^ with anionic ligands as chloride (Cl-), inorganic sulfide, and thiols (-SH)^91^. This binding could be resulted in reduction in the bioavailability of Ag^+^ for destroying cells^5,92^. Moreover, some structural as well as functional modifications could be seen in proteins and antioxidants^93^. According to Garner *et al*.^94^ great reduction of glutathione (RBC intracellular antioxidant) at exposing erythrocytes to 30 nm silver colloids. The nanosilver toxicity of RBCs might be due to free silver ions release, total silver ion concentration and/or interaction between cellular components and nanoparticles^6,92^. On the other hand, there was always release of silver ions when AgNPs came in contact with the RBCs^95^. This leakage of silver ions may not be altered by joining to either transmembrane proteins or plasma chloride. In addition, nonspecific AgNPs transport into RBCs was found to take place depending on particles size (≤200 nm) and not regulated by the type of particle material or charge. Finally, it concluded that in addition to production of silver ions, decomposition, binding as well as membrane vesiculation may be the mechanisms responsible for induction of hemolysis.

### Cytotoxicity of silver nanoparticles

**Figure 10:**
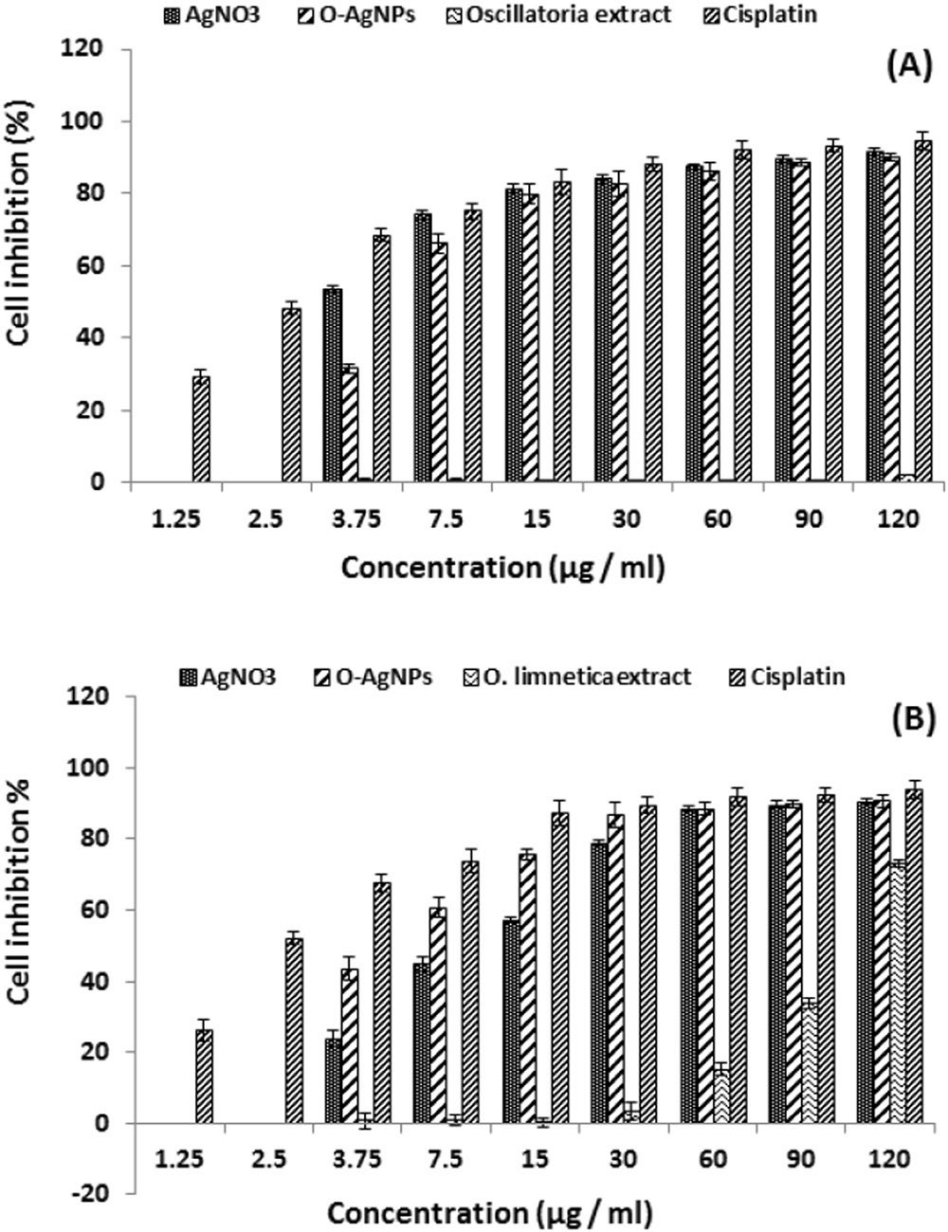
Cytotoxicity of O-AgNPs on human breast cancer cell line (M CF-7) (**A**) and colon cancer cell line (HCT-116) (**B**).

**Figure 11:**
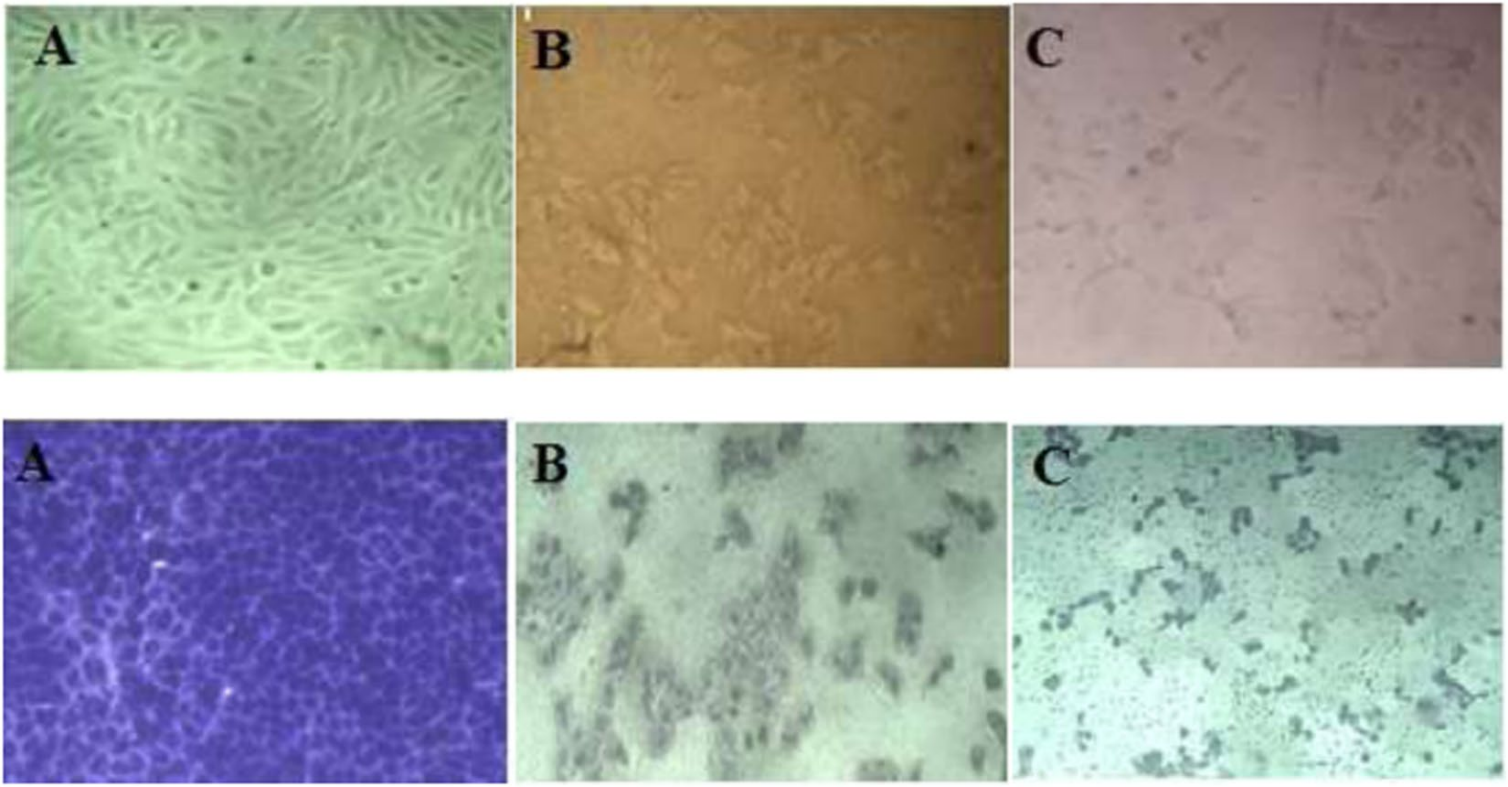
Microscopic evaluation of the effect of the *O. limnetica* extract (**A**), O-AgNPs (**B**) as well as the silver nitrate alone (**C**) in a bright field (top raw) or after staining with crystal violet (lower raw) on MCF-7 cells at concentration of 120 µg/ml.

Cancer is a significant reason for mortality worldwide. The biological role of silver nanoparticles in diagnosis and bioremediation of human cancers gained a great interest nowadays. In this study, the Cytotoxicity of O-AgNPs was evaluated against two human cancer cell lines (MCF-7 & HCT-116) using MTT assay *in vitro*. O-AgNPs exhibited cytotoxic effect against MCF-7 & HCT-116 cell lines in dose dependent responding manner attending the following descending order 120 > 90 > 60 > 30 > 15 > 7.5 > 3.5 µg/ml, whereas the cytotoxic action (IC50) was observed at 6.147 µg/ml against MCF-7 and 5.369 µg/ml for HCT-116 cells (Fig. 10). Consequently, the prepared O-AgNPs demonstrated potent cytotoxic action against HCT-16 more than MCF-7 cell lines. O-AgNPs treatment induced apoptosis represented by changes in the morphology of cells (Fig. 11). IC50 of AgNO_3_ addition recorded as 2.64 µg/ml and 11.12 µg/ml against MCF-7 and HCT-116 respectively. In general data revealed that *O*. *limnetica* extract had no inhibitory effect on MCF-7 cell line, whereas high concentrations have an inhibitory effect on HCT-116 cell line. Cisplatin Cytotoxicity quantified as 2.61 µg/ml and 2.43 µg/ml for MCF-7 and HCT-116 cell lines respectively which was generally comparable to the cytotoxic effect of the biosynthesized nanoparticles. The penetration of nanoparticles into the mammalian cells via either phagocytosis or endocytosis significantly dependent on the minute size of the AgNPs^96^. Nanoparticles characterized by size ranged between 30 to 100 nm cannot penetrate deeply through the tumor matrix depositing on the wall of the blood vessel and consequently cannot reach the tumor cells away from the blood vessels, whereas surface charge and the small size of AgNPs encouraged good diffusion and dispersion into tumor matrix^97^. Numerous attempts have been documented to elucidate the mechanism of action of nanoparticles cytotoxicity via the manufacture of free radicals, which cause cellular disintegration leading to cell death^98,99^. Many studies has been documented the anticancer potentiality of AgNPs through their ability to scavenge the free radicals^21,100,101^. Rahman *et al*.^102^ suggested that the absorbed AgNPs would frequently attained the present free electrons resulting increments in synthesis of reactive oxygen species (ROS). Consequently, this increase in synthesis as well as the accumulation of damaging radicals which attack protein causing oxidative stress would be resulted in partial or permanent damage of protein integrity and functionality. Its also found that AgNPs regulate the DNA-dependent kinase activity which takes place in repairing DNA damage^103^. Various sensitivity of breast cancer cells to silver nanoparticles causing hyperthermia, by which AgNPs may be considered as successful photo thermal treatment^104^. The current results are in accordance of Ranijitham *et al*.^105^ where they reported that the detected cytotoxic effect against MCF-7 cell line responded positively with different AgNPs concentrations in dose- response manner.

## Materials and Methods

### *Oscillatoria limnetica* and culture conditions

Cyanobacterium, *Oscillatoria limnetica* was selected from the algal culture collection of phycology lab, faculty of science, Mansoura University. *O*. *limnetica* axenic inoculum was cultivated into 500 ml Erlenmeyer flasks containing 200 ml BG11 medium^106,107^. The flasks were grown under continuous illumination (57.75 M mol m^−2^ s^−1^) at 28 ± 1 °C) for 22 days where the beginning of the stationary growth phase was reached.

### Preparation of algal extract

200 ml of *O*. *limnetica* culture was centrifuged for 10 min at 1610 × g using Centurion Scientific, United Kingdom centrifuge. The resulted biomass pellet was added to 10 ml deionized water and sonicated for 15 min at 100 amplitude using Ultrasonic Homogenizer (Cole-Parmer Instrumental Co. Chicago, Illinois 66648 USA). The obtained homogenate was completed to 100 ml with deionized water and stored at 4 °C for further use. The corresponding dry weight of the used biomass was determined (0.154 g dry biomass/L). Flavonoids were determined in the crude extract according to the method of Farasat *et al*.^108^.

### Extracellular biosynthesis and optimization of oscillatoria-silver nano scale particles

For the synthesis of O-AgNPs, 5 ml of the previously prepared *O*. *limnetica* homogenate was completed to 19 ml by 0.1 M phosphate buffer at pH 7, and then 1 ml silver nitrate solutions (0.1, 0.2, 0.3, 0.4 & 0.5 mM) was added separately to detect the most effective AgNO_3_ concentration. The reaction mixture was incubated at 35 ± 1 °C under illumination (75.9 µmol m^−2^ s^−1^). The original color of the mixture was greenish converted to dark brown, indicating the formation of O-AgNPs. The bioreduction of silver ions was monitored by scan spectrophotometer (Jenwayuv/visible-2605 spectrophotometer, England) every 6 hours intervals for 48 hours in order to investigate time course of the process. Then, the biofabricated O-AgNPs were collected by centrifugation (MIKRO 12 Hettich Zentrifugen D-78532 Tuttlingen-Germany) at 4528 × g for 10 minutes, then washed with deionized water, dried and stored at room temperature. For optimizing the AgNPs biosynthesis process, the following algal homogenate concentrations were used 1, 2, 3, 4, 5 & 6 ml as well as the effect of pH was studied by adjusting pH of the reaction mixture to 4.7, 5.7, 6.7, 7.7, and 8.7. In addition UV-Vis spectrum of blank (AgNO_3_ solution and *O*. *limnetica* extract without AgNO_3_) was measured. Time-Dependent stability of O-AgNPs preparation was conducted for 9 months using scan UV-visible spectroscopy where the preparation was stored under dark conditions in closed glass for 1 month, 6 months and 9 months.

### Characterization of O-AgNPs

The biosynthesis of silver nanoparticles was scanned in the range of 200–900 nm using UV-vis spectrophotometer (ATI Unicam 5625 UV/VIS Vision Software V3.20) at Spectrum Unit of Faculty of Science, Mansoura University, Egypt.

Fourier transforms infrared (FT-IR) was used to study to investigate the chemical functional groups of *O*. *limnetica* fresh biomass involved in the bioreduction of silver nanoparticles (ThermoFisher Nicolete IS10, USA) in the region of 4000–500 cm^−1^ at a resolution of 1 cm^−1^. The particle size, shape and morphology of the biosynthesized O-AgNPs were characterized by transmission electron microscopy (JEOL, JEM-2100, Japan), as well as the surface characteristics using scanning electron microscope (JEOL JSM-6510/v, Japan).

### Bactericidal potentiality of O-AgNPs

Bacteria selected for this investigation are two human pathogens, one of them is the *Escherichia coli* (Gram^−^) and the other is *Bacillus cereus* (Gram+) and they were kindly supplied by bacteriology lab, faculty of science, Mansoura University.

#### Bacterial growth inhibition (using luria-bertani broth medium)

To investigate the bactericidal activity of the synthesized O-AgNPs, growth inhibition study was conducted. An aliquot (500 µl) of O-AgNPs preparation was supplemented to 9 ml of LB (Luria-Bertani) medium and inoculated with 500 µl of the studied bacterial suspensions (1 × 105 CFU ml^−1^) separately and incubated at 37 °C for 24 h using shaker incubator (New Brunswick Innova 4330 Refrigerated Incubator Shaker) adjusted to 150 rpm. All treatments were performed in triplicates. Control cultures were supplemented with 500 µl LB medium instead of O-AgNPs preparation. Cefaxone (broad spectrum antibiotic), AgNO_3_ solution and aqueous extract of *O*. *limnetica* fresh biomass were added for comparison as previously mentioned. Bacterial growth was determined by measuring the turbidity of the bacterial cultures using UNICO UV-2000 spectrophotometer, at 600 nm. The bacterial growth inhibition was calculated as adopted by Banjara *et al*.^109^ as following:

$${\rm{Percentage}}\,{\rm{of}}\,{\rm{growth}}\,{\rm{inhibition}}=\frac{({\rm{OD}}\,{\rm{of}}\,{\rm{control}}-{\rm{OD}}\,{\rm{of}}\,{\rm{test}})}{{\rm{OD}}\,{\rm{of}}\,{\rm{control}}}\ast 100$$

The changes in the structural characteristics of *Bacillus cereus* (Gram+) was detected by transmission electron microscopy (TEM) for both control and treated cultures in addition to investigate the chemical composition of the bacteria treated with O-AgNPs using the energy-dispersive X-ray spectroscopy (EDX) detector operated at an accelerating voltage of 20 kV to perform elemental analysis (JEOL, JEM-2100, and Japan) at the Electron Microscope Unit, Mansoura University, Egypt.

### Antibacterial potentiality of O-AgNPs by disk-diffusion method

Antibacterial potential was evaluated in response to the previously mentioned pathogenic bacterial strains by the disk diffusion test, using antibiotic saturated disks^110^. The test was conducted by using filter paper (Whattman No. 3) discs (6 mm in diameter) impregnated with 50 μl of the test solutions that was placed over the inoculated medium surface. Formation of antibiotic-OAgNPs conjugates was prepared according to Harshiny *et al*.^111^, where equal volumes of O-AgNPs (10 mg/ml^−1^) and antibiotic (10 mg/ml^−1^) were mixed using phosphate buffer and incubated at room temperature for 24 h. Then the mixture was centrifuged (10000 rpm) for 10 min. Cefaxone (10 mg/ml^−1^), Tetracycline (10 mg/ml^−1^), Cefaxone-conjugated O-AgNPs, Tetracycline-conjugated O-AgNPs, aqueous extract of *O*. *limnetica* and AgNO_3_ solutions as well were also used as reference for antimicrobial agents, and the plates were incubated at 37 °C for 24 h after which they were examined for the existence of inhibition zones. The diameter of the inhibition zones was taken as functions of antibacterial potentiality were expressed in millimeter.

### Hemolytic assay of oscillatoria-silver nano scale particles

Toxic potential and/or cell lysis capacity of the synthesized O-AgNPs was investigated according to Surendra *et al*.^112^. Human blood sample was centrifuged for 10 min at 226 × g to obtain the red blood corpuscles pellet which was purified by repeating the process three times by using PBS (phosphate-buffered saline) at pH 7.4. Aliquots of red blood corpuscles (100 μL) were added to 100 μL of O-AgNPs preparation containing different concentrations (2.5, 5.0, 7.5 and 10 μg/ml) and the final volume was completed to 1 ml using PBS and incubated for 60 min at room temperature. Then, after centrifuging the sample at 226 × g for 10 min, the absorbance of the supernatant was estimated at 540 nm. For verifying the hemolysis assay, positive and negative controls were prepared from Triton-X 100 (TTX-100) and blood sample with PBS, respectively in triplicates. Hemolysis percentage was computed from the following equation,

$$({\rm{As}}-{\rm{Anc}}/{\rm{Apc}}-{\rm{Anc}})\ast 100$$

where, as is the absorbance of sample, Anc is the absorbance of negative control, Apc is the absorbance of positive control.

### Antitumor activity of oscillatoria-AgNPs *in vitro*

#### Cell lines

Mammary gland breast cancer cell line (MCF-7) and colon cancer cell line (HCT-116) were supplied from the American Type Culture Collection (ATCC) through the Holding company for biological products and vaccines (VACSERA), Cairo, Egypt. The cells were maintained at 37 °C and 5% CO_2_ in DMEM (Lonza, 12-604 F) medium supplemented with 5% fetal bovine serum (FBS, Lonza, Cat. No. 14-801E), 100 IU/ml penicillin and 100 µg/ml streptomycin (Lonza, 17-602E).

### Cytotoxicity assay

The cytotoxic effect of the biosynthesized O-AgNPs was assessed *in vitro* using the standard MTT [3-(4, 5-Dimethyl thiazol-2yl)-2, 5-diphenyl tetrazolium bromide] reduction assay on MCF-7 and HCT-116 cell lines^113^. Colorimetric MTT assay based on the production of purple crystals, derived from the splitting action of NAD-dependent mitochondrial dehydrogenase on the yellow colored solution of tetrazolium salt MTT in phosphate buffered saline (PBS) which led to the formation of formazan crystals that represent a function of positive correlation with the viable cell count and contrary proportional to the Cytotoxicity level. The cells were seeded in 96-well plates as 5 * 104 cells/mL (100 µL/well) and exposed to different concentrations of O-AgNPs as well as algal extract and AgNO_3_ in the following concentrations for each: 3.75, 7.5, 15, 30, 60, 90, 120 µg/ml. Then incubated at 37 °C and supplied with 5% CO_2_ for 48 hrs. After that, 15 µl of MTT solution (5 mg/mL PBS) was added to each well and incubated for another 4 hours. The formazan crystals were solubilized by 100 µL of DMSO and the color developed was measured at 570 nm using a Bio-Tek plate reader (ELx 800, USA). Each experiment was repeated three times and standard deviation was calculated. The percentage of relative cell viability was calculated as (A570 of treated samples/A570 of untreated sample) X100. IC50% was calculated as the concentration that causes 50% inhibition for cell growth after 48 hrs of incubation, compared with untreated cells. The growth of the cells were monitored and the images were acquired by Gx microscopes (GXMGXD202 Inverted Microscope) (10x Eyepiece) after staining with crystal violet^114^. Cisplatin was used as a standard anticancer drug (positive control). This study was conducted in Liver Cancer Center of Pharmacognosy department in faculty of pharmacy – Mansoura University.

### Statistical analysis

Data were subjected to analysis of variance and the means were compared using the “Least Significant Differences (LSD)” test at the 0.05 level, as recommended by Snedecor *et al*.^115^.

## Conclusion

This study indicated that the green synthesized silver nanoparticles via simple biological protocol using *Oscillatoria limnetica* aqueous extract that supplied both reducing and stabilizing agent for the biosynthesis of nanoparticles where the extracellular biosynthesis of O-AgNPs facilitates the process for downstream processing. The biosynthesized O-AgNPs have a synergetic bactericidal potential (accompanied with antibiotics) advantages as biocontrol mediators for some human pathogenic bacteria (*E*. *coli* and *B*. *cereus*) according to its stability and minor size. In addition O-AgNPs exhibited low haemolytic activity may be useful in administration of some medical devices as well as having cytotoxic action on some human cell lines (breast (MCF-7) cell line and human colon cancer (HCT-116). O-AgNPs characterization may be introduced a promising applications in medicine, cosmetic and pharmaceutical industries.
